# Successful Transcatheter Closure of a Rare Malaligned Atrial Septal Defect With a Membranous Chord

**DOI:** 10.1016/j.jaccas.2021.05.024

**Published:** 2021-08-19

**Authors:** Syed W. Haider, Apurva Patel, Edgar Argulian, Tak W. Kwan

**Affiliations:** Division of Cardiology, Icahn School of Medicine, Mount Sinai Morningside Hospital, New York, New York, USA

**Keywords:** atrial septal defect, intracardiac echocardiography, malaligned atrial septal defect, transcatheter closure, ASD, atrial septal defect, ASO, atrial septal occluder, CCT, cardiac computerized tomography, ICE, intracardiac echocardiography, LUPV, left upper pulmonary vein, RLPV, right lower pulmonary vein, TCC, transcatheter closure, TEE, transesophageal echocardiogram, TTE, transthoracic echocardiogram

## Abstract

Atrial septal defect (ASD) is a common congenital defect that leads to various hemodynamic complications if untreated. Transcatheter closure (TCC) of isolated secundum ASD is the preferred treatment. Herein we describe a unique malaligned ASD secondary to a membranous chord. With balloon sizing and intracardiac echocardiography (ICE), TCC was successfully pursued. (**Level of Difficulty: Beginner.**)

## Central Illustration

**Figure:**
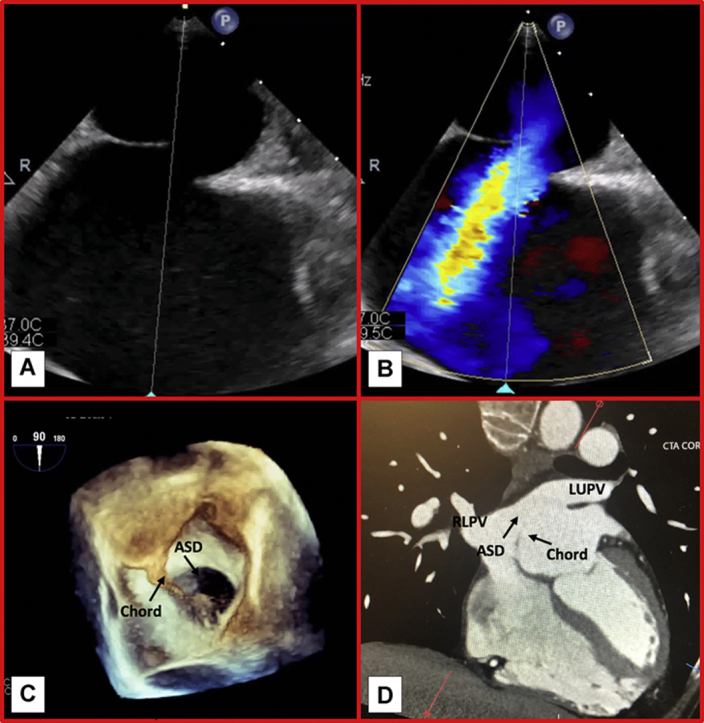

## History of Presentation

A 59-year-old man presented with atypical chest pain, orthopnea, and bilateral leg swelling, progressively worsening in the past 6 months. On examination, a soft systolic ejection murmur was heard at the left second intercostal space. He also had 1+ lower extremity bilateral pitting edema up to the level of his shins. The remainder of the examination results were unremarkable. The vital signs and laboratory test results were within normal limits.

**Learning Objectives**

• To understand anatomical associations in septum secundum ASD leading to malalignment.
• To explore outcome of ASD related to septal malalignment with TCC.
• To use ICE and balloon sizing in ASD closure.

## Past Medical History

The patient had smoked one pack of cigarettes daily for the past 10 years. He took no medications and had no prior surgeries or hospital admissions.

## Differential Diagnosis

The differential diagnoses included congestive heart failure, coronary ischemia, and congenital heart defect.

## Investigations

**Figure 1:**
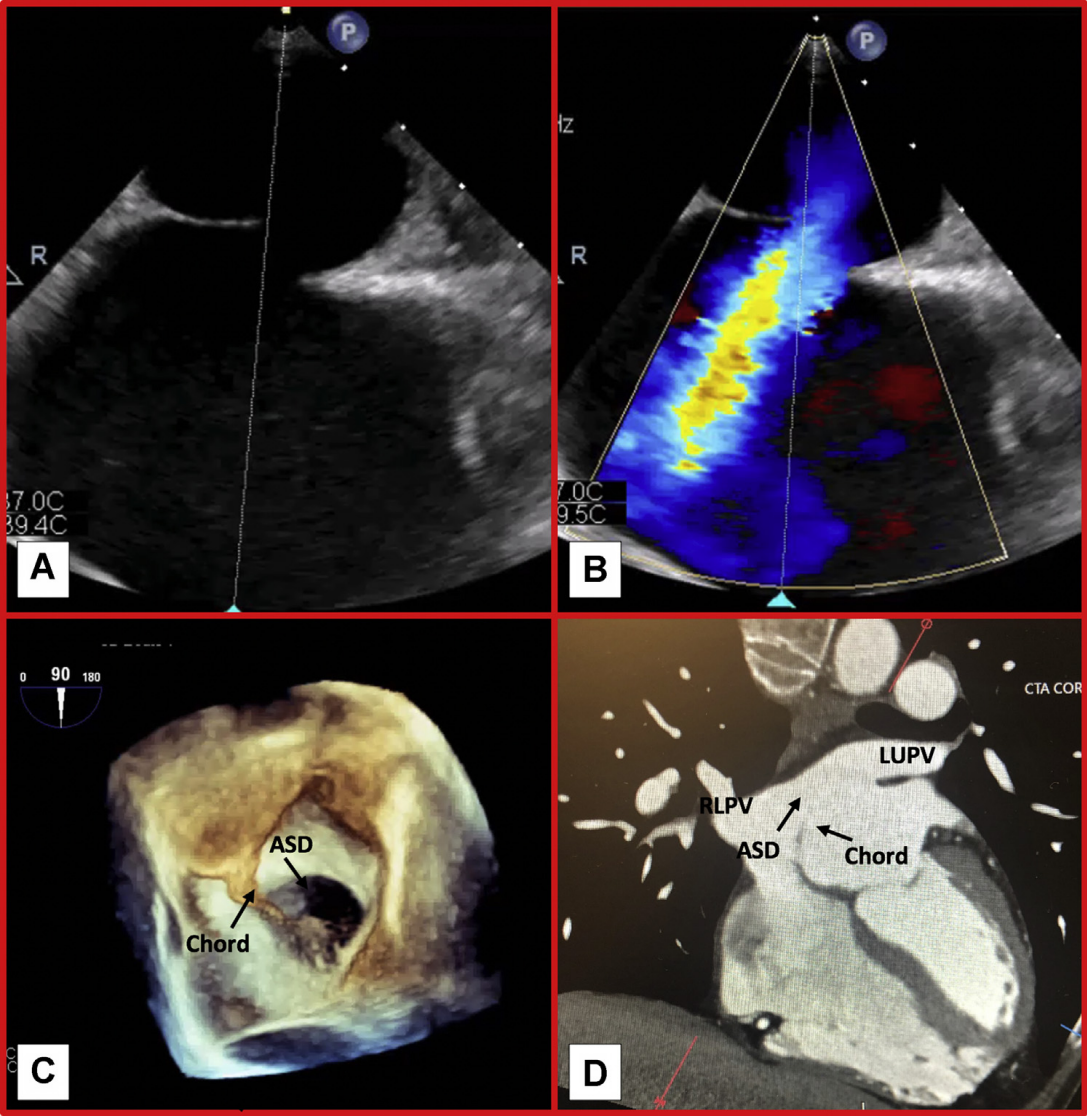
Tranesophageal Echocardiogram and Cardiac Computerized Tomography Findings Two-dimensional transesophageal echocardiography views with **(B)** and without **(A)** color Doppler revealing the malaligned atrial septal defect (ASD). **(C)** 3-Dimensional image of left atrial perspective of ASD along with the membranous chord. **(D)** Cardiac computerized tomography confirming attachment of membranous chord to the left atrial wall. LUPV = left upper pulmonary vein; RLPV = right lower pulmonary vein.

A TTE was initially performed, which revealed preserved left ventricular function and size. The RV was mildly dilated with preserved function. The Doppler study showed moderate tricuspid regurgitation, and the other valves were normal. A bubble study with agitated saline injection showed right-to-left flow suggestive of an intracardiac shunt. A TEE was subsequently performed. A defect was seen in the interatrial septum measuring 2.37 cm × 0.97 cm, consistent with a secundum-type ASD. Doppler study showed low-velocity left-to-right atrial flow. Interestingly, a thin membranous chord was also noted, seen pulling the inferior rim of the defect and causing an evident septal malalignment (Figure 1, Videos 1 and 2). Off-axis TEE views revealed the chord to be attached to the superior-anterior portion of the left atrium. Measurements of rims were as follows: superior rim, 0.95 cm; inferior rim, 1.54 cm; aortic rim, 0.9 cm. Pulmonary venous attachments were normal. CCT was performed, given the atypical chest pain, and showed no calcified coronary or aortic plaque and a calcium score of 0. The CCT further confirmed normal pulmonary venous attachments and no concomitant congenital abnormality. Finally, the CCT confirmed the chord to be attached to the left atrial wall, not to any other cardiac structure that could lead to complications should iatrogenic resection occur (Figure 1).

## Management

The structural interventional team was consulted for consideration of TCC of ASD. The septal malalignment secondary to the membranous chord was considered a potential barrier to the success of TCC. After the risks and benefits of TCC versus surgery were discussed with the patient, TCC was decided.

**Figure 2:**
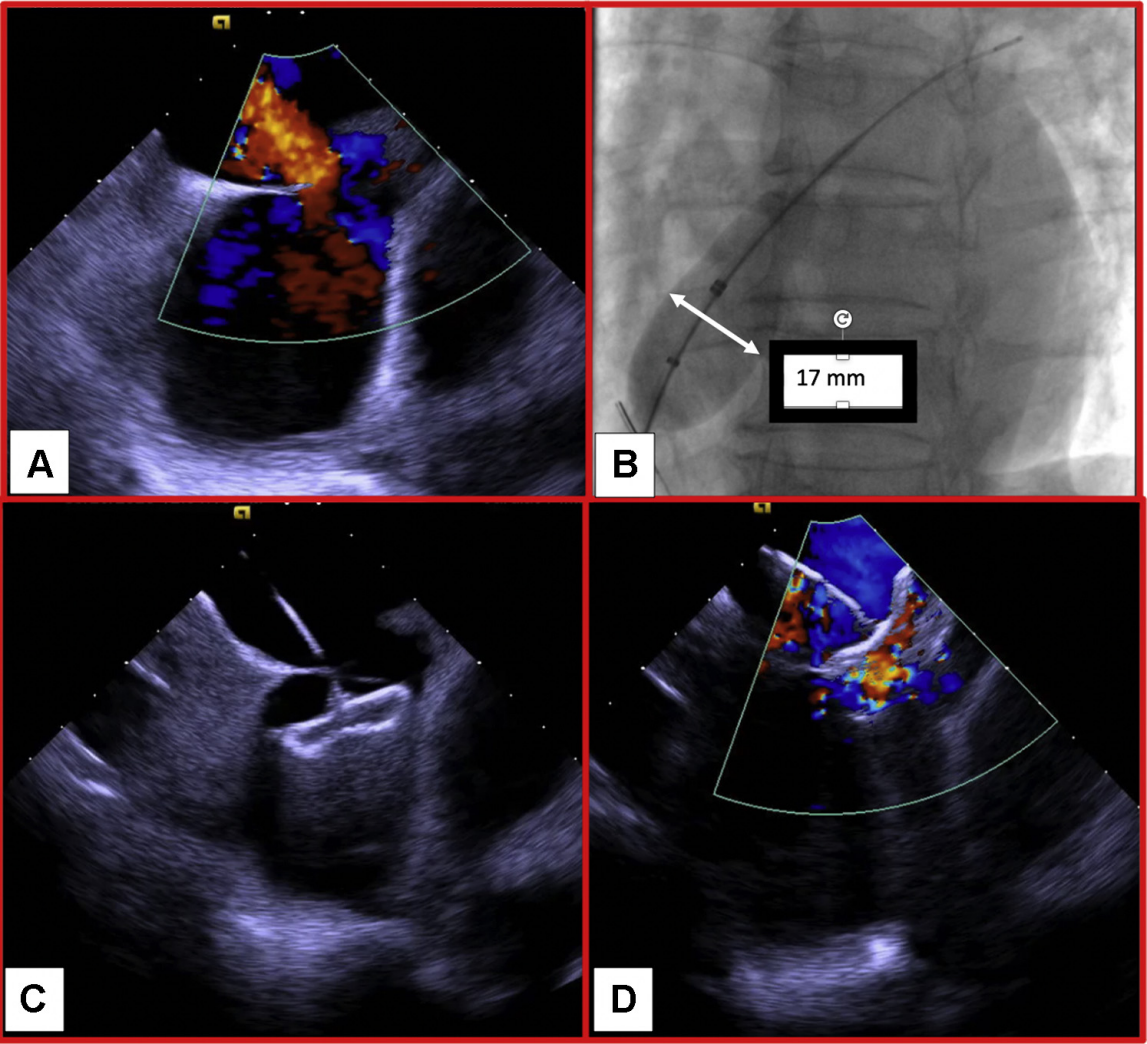
Intra-Operative Findings on Intracardiac Echocardiography **(A)** Intracardiac echocardiography showing left-to-right flow through malaligned atrial septal defect. **(B)** Fluoroscopic guided balloon-sizing of atrial septal defect. Intracardiac echocardiography images without **(C)** and with **(D)** color Doppler showing atrial septal occluder deployment.

**Figure 3:**
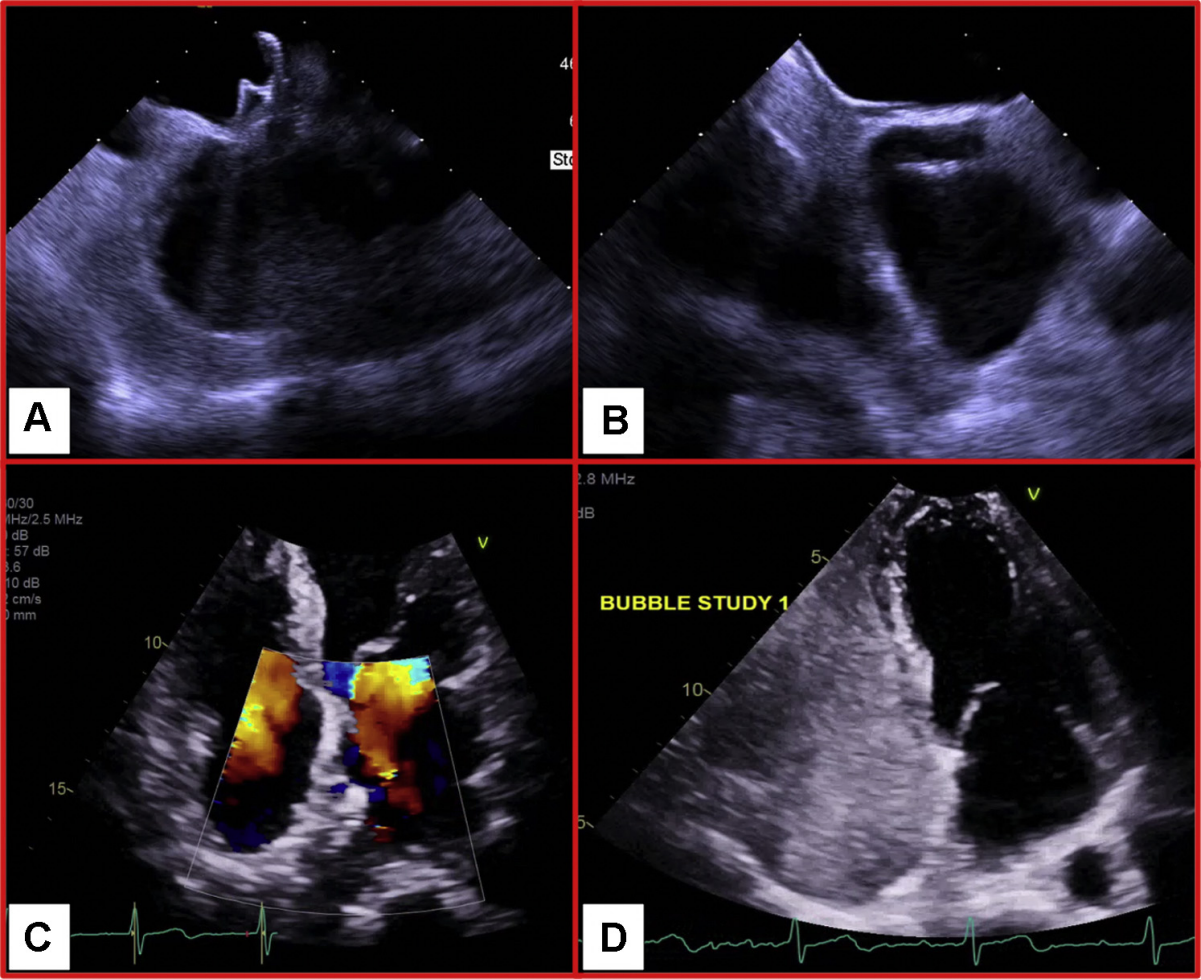
Post-Deployment Results **(A, B)** Post-deployment intracardiac echocardiography images showing atrial septal occluder to be adequately sandwiched in the atrial septum. **(C)** Transesophageal echocardiography on postoperative day 1 showing trivial interatrial doppler flow through the septum. **(D)** Bubble study with agitated saline showing no intracardiac shunt.

ICE was used for sizing of the defect, measuring up to 17 mm × 14 mm (Figure 2, Video 3). Balloon sizing was performed under fluoroscopic guidance, and the defect was estimated to be approximately 17 mm. To ensure adequate approximation of the malaligned defect, a decision was made to slightly oversize the device with a 20-mm ASO. After deployment of the ASO (Video 4), no residual interatrial shunt was seen. A tug test showed the device to be well-seated. Of important note, the membranous chord appeared “sandwiched” between the ASO and remained intact. TTE the next day showed adequate sandwiching of the septum with ASO without any pericardial effusion. A bubble study did not show any evidence of right-to-left shunt with Valsalva maneuver (Figure 3). The patient was discharged home the next day, to take aspirin 81 mg daily indefinitely and clopidogrel 75 mg daily for 1 month.

## Follow-Up

At his 1-month follow-up visit, the patient appeared euvolemic and with improvement in symptoms. A repeated TTE showed improvement in RV size. The ASO was well seated. No intracardiac shunt was seen with agitated saline contrast injection.

## Discussion

ASD is a common congenital cardiac defect, with a reported incidence of 1 per 1,000 live births (1). Untreated, it can lead to right ventricular overload with heart failure, atrial arrhythmias, pulmonary hypertension, and premature death (2). Ostium secundum defect occurs when there is increased resorption of septum primum tissue in the atrium's roof, or if the septum secundum does not occlude the ostium secundum. The membranous chord in the current case may represent membranous tissue formation after migration of the mesenchymal cells with failed resorption. Septal malalignment is relatively common, reported to be 44% in one study (3). Previously, TCC of a malaligned patent foramen ovale secondary to an atrial chord has been described (4). However, to our knowledge, the current case is the first to report septal malalignment of a secundum-type ASD caused by a membranous chord that underwent successful TCC (3).

TCC of isolated secundum ASD has emerged as an effective alternative to surgery (5). However, serious complications such as device erosion and embolization may occur with deficient aortic rim and incorrect device sizing (6). Recently, studies have found a strong association of septal malalignment with device complications (4,5). In septal malalignment, the septum primum is malaligned toward the left atrial aspect and is separated from the septum secundum. At the time of TCC, septal malalignment can alter the device axis angle against the aorta, potentially pushing the device disc to the aorta with attendant device instability. To mitigate these complications, it is crucial to delineate the anatomy of the septal malalignment with possible modifications in technique.

In the current report, ASD closure was indicated owing to right ventricular dilation (7). However, given the complexity of this malaligned ASD, close attention was paid to appropriate device sizing with ICE. ICE has gradually replaced TEE guidance in TCC of ASD (8). TEE provides high-resolution images, but it warrants adequate sedation. ICE, however, can be used with minimal to no sedation but requires venous access that may be associated with venous complications (9). We used both ICE and balloon-guided technique for defect sizing, which is the new standard of care. The recommendation to give the patient 1 month of dual antiplatelet therapy is also in line with contemporary practice.

## Conclusions

Optimal echocardiographic assessment and TEE views—both standard and off axis—are needed to fully evaluate the ASD anatomy and rule out other associated defects. TCC is standard of care for secundum ASD, but the morphologic spectrum and associated abnormalities such as the membranous chord in this case can influence the outcome of TCC. ICE-guided TCC along with balloon sizing of the defect is an excellent modality for ensuring optimal results in ASD closure, especially when other embryologic defects such as atrial chords are seen.

## Funding Support and Author Disclosures

The authors have reported that they have no relationships relevant to the contents of this paper to disclose.

## Appendix

Supplemental Video 1 3D TEE images of left atrial perspective of ASD along with the membranous chord.

Supplemental Video 2 TEE X-plane imaging without and with color Doppler revealing malaligned ASD and left-to-right flow.

Supplemental Video 3 ICE images without and with Doppler during ASO deployment.

Supplemental Video 4 Post-deployment ICE images
